# Identification of Bacterial Wilt (*Erwinia tracheiphila*) Resistances in USDA Melon Collection

**DOI:** 10.3390/plants10091972

**Published:** 2021-09-21

**Authors:** Bimala Acharya, Lucas Mackasmiel, Ali Taheri, Christine A. Ondzighi-Assoume, Yiqun Weng, C. Korsi Dumenyo

**Affiliations:** 1 Department of Agricultural and Environmental Sciences, Tennessee State University, Nashville, TN 37209, USA; bacharya@my.tnstate.edu (B.A.); lmackasm@Tnstate.edu (L.M.); ataheri1@tnstate.edu (A.T.); condzigh@tnstate.edu (C.A.O.-A.); 2 USDA-ARS, Vegetable Crops Research Unit, Horticulture Department, University of Wisconsin, Madison, WI 53706, USA; yiqun.weng@usda.gov

**Keywords:** melon, bacteria wilt, *Erwinia* *tracheiphila*, fluorescent microscopy, disease resistance, germplasm screening

## Abstract

Bacterial wilt (BW) caused by the Gram-negative bacterium, *Erwinia tracheiphila* (Et.), is an important disease in melon (*Cucumis melo* L.). BW-resistant commercial melon varieties are not widely available. There are also no effective pathogen-based disease management strategies as BW-infected plants ultimately die. The purpose of this study is to identify BW-resistant melon accessions in the United States Department of Agriculture (USDA) collection. We tested 118 melon accessions in two inoculation trials under controlled environments. Four-week-old seedlings of test materials were mechanically inoculated with the fluorescently (GFP) labeled or unlabeled *E. tracheiphila* strain, Hca1-5N. We recorded the number of days to wilting of inoculated leaf (DWIL), days to wilting of whole plant (DWWP) and days to death of the plant (DDP). We identified four melon lines with high resistance to BW inoculation based on all three parameters. Fluorescent microscopy was used to visualize the host colonization dynamics of labeled bacteria from the point of inoculation into petioles, stem and roots in resistant and susceptible melon accessions, which provides an insight into possible mechanisms of BW resistance in melon. The resistant melon lines identified from this study could be valuable resistance sources for breeding of BW resistance as well as the study of cucurbit—*E. tracheiphila* interactions.

## 1. Introduction

Cucurbit crops, belonging to the family Cucurbitaceae, are grown widely in both tropical and subtropical regions of the world [1,2]. The Cucurbitaceae family comprises 96 genera and about 1000 species, out of which 33 are cultivated species including major cucurbits such as cucumber (*Cucumis sativus* L.), melon (*Cucumis melo* L.), watermelon (*Citrullus lanatus* Thunb.) and pumpkin/squash (*Cucurbita* spp. L.) [3]. Cucurbits are grown mostly as fruits and vegetables for human nutrition. Some cucurbits are consumed raw as desserts (watermelon, muskmelon) and salad (cucumber and long melons), while some others are cooked as vegetables (bottle gourd, bitter gourd, sponge gourd, ridge gourd, summer squash, squash melon, pumpkin, etc.). After post-harvest processing, some cucurbits such as cucumber and pointed gourd are used as pickles, while pumpkin and ash gourd are used in jam and candy industries, respectively [4]. In the United States, melons are cultivated on approximately 300,000 acres annually with equivalent to over two million tons annual output from 2017 to 2020 [5].

Cucurbits are afflicted by more than 200 infectious plant diseases caused by fungi, bacteria, viruses or phytoplasmas [6]. In the United States, bacterial wilt (BW) is among the most devastating diseases for cucurbits that may cause up to 80% of yield loss in susceptible varieties [7,8]. Among different cucurbits, cucumber and melon are the most susceptible to BW, while watermelon is generally resistant [9,10,11].

Bacterial wilt is caused by the Gram-negative bacterial pathogen, *Erwinia tracheiphila*, which is vectored by the striped cucumber beetle (*Acalymma vittatum*) and spotted cucumber beetle (*Diabrotica undecimpunctata*). The bacteria overwinter in the gut of the beetles and are transmitted through the feeding and frass [12,13,14,15]. Inside the plant, the bacterium produces extracellular polysaccharides and blocks fluid flow in the xylem through the mechanism of vascular occlusion causing the plant to wilt [16]. There is presently no effective pathogen-based disease management strategy for control of BW, and infected plants will ultimately die. Alternate strategies target the vectors. Cultural practices for the management of the vectors include crop rotation or intercropping with non-cucurbit crops, transplanting rather than direct seeding, use of floating row covers, perimeter trap crops and straw or reflective plastic mulches [17]. Chemically, there are options such as the use of kaolin clay, pyrethrum, spinosad and plant extracts such as neem oil and inducers of systemic acquired resistance for the management of the disease or the beetle vector. However, results from field trials with these organic insecticides have been inconsistent [18]. Chemical insecticides, therefore, remain the predominant weapon against cucumber beetles that are commonly used in BW management especially in commercial production. However, broad-spectrum insecticides have adverse effects on the environment and beneficial insects such as pollinator species [19].

Deployment of host resistance is a key component in any integrated pest management strategy. However, there is no widespread use of resistant melon and cucumber varieties in commercial production. Additionally, surprisingly, despite the importance of BW in melon and cucumber production, a literature review found very little information on the utilization of host BW resistance in these two important vegetable crops. The United States Department of Agriculture (USDA) Germplasm Resource Information Network (https://www.ars-grin.gov/; accessed on 17 September 2021) has a large collection of melon (>2000 accessions) and cucumber (~1300 accessions), which have played critical roles in US melon and cucumber breeding by providing important disease resistance sources. However, limited work has been done to screen the melon and cucumber germplasm for resistance to bacterial wilt [20,21], which is the first step in developing BW-resistant varieties [22,23]. Thus, the objective of this study was to screen melon accessions for resistance to bacterial wilt disease in response to mechanical inoculation by bacterial wilt pathogen, *Erwinia tracheiphila*. Using fluorescence microscopy, we also observed the dynamics of *E. tracheiphila* pathogen development in resistant and susceptible melon lines.

## 2. Results

### 2.1. Identification of Melon Lines for BW Resistance

**Figure 1:**
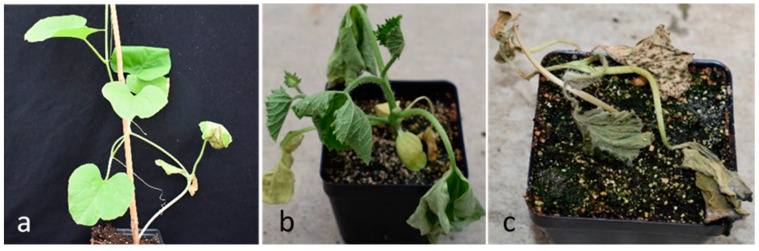
Development of symptoms of bacteria wilt in melon. Days to wilting of inoculated leaf (DWIL), symptom: Only the inoculated leaf wilts (**a**), days to wilting of whole plant (DWWP), symptom: the whole plant wilts (**b**), days to death of whole plant (DDP), symptom: whole plant wilts and dries (**c**).

To identify BW resistant melon lines, we screened 118 melon accessions for resistance to mechanical inoculation with the *E. tracheiphila* strain Hca1-5N. For comparison, we also included five cucumber accession in the experiments. The complete names of these accessions, their taxonomic status and geographic origins are presented (Supplemental Table S1). The screenings were performed in two greenhouse experiments conducted in summer 2019 (EXPT1) and autumn 2019 (EXPT2), respectively. Due to space limitation, we tested 64 lines (61 melon and three cucumber lines) in both experiments. Additionally, 57 melon and two cucumber lines were tested in either summer or autumn 2019 trial alone. From the day of inoculation, the seedlings were observed daily for symptom development for one month. Data were recorded for days to wilting of the inoculated leaf (DWIL), days to wilting of the whole plant (DWWP) and days to death of the plant (DDP). Typical symptoms of BW disease after inoculation are shown in Figure 1.

The mean values of DWIL, DWWP and DDP for 64 melon and cucumber lines tested in both 2019 Summer and Autumn trials are provided in Table 1, and those tested in only one season are presented in Table 2. Statistical analyses were performed to test these mean values with one-way ANOVA (*p* < 0.05). The DWIL gives an idea of how long it takes for a plant to show the first symptom of disease, wilting of inoculated leaf after inoculation. Similarly, the record of DWWP pictures the progression of disease and DDP gives the duration of survival of plant after inoculation. Accessions with significantly higher value for these parameters suggested better resistance (survival ability) to BW infection than those with lower values. For each parameter, an accession is considered resistant if its mean is followed by the letter “a” in Tukey HSD analysis. Those means rank at the highest category of means for that parameter. The accessions that did not show resistance in any of the three parameters were classified as susceptible (S). Resistance in one parameter was classified as low (L), two parameters as medium (M) and three parameters as high (H) in at least one of the experiments. It was clear that there was significant variation among the test lines for resistance against *E. tracheiphila* infection (Table 1 and Table 2; Figure 1). Of the 23 accessions that had a score of low resistance, 15 were screened in both experiments and eight in one experiment (Table 3). There were six accessions that had medium resistance, four were screened in both experiments and two in one experiment. Importantly, four melon accessions, Ames 13299, PI 370441, PI 230186 and PI 200814, had significantly higher scores for DWIL, DWWP and DDP suggesting high BW resistance. Although the disease resistance ratings of high (H), medium (M) or low (L) were based on the performance of that accession in any one of the two experiments, it was interesting to observe that the first three lines in Table 1 with a resistance rating of H (Ames 13299, PI 200814 and PI 230186) also had a resistant rating in two parameters, while PI 370441 had a resistance rating in one parameter in the second experiment. Overall, of the 90 susceptible accessions, 41 were validated in both experiments and 49 in one experiment (Table 3).

### 2.2. Localization of the Bacterium and Colonization in the Melon Host

**Figure 2:**
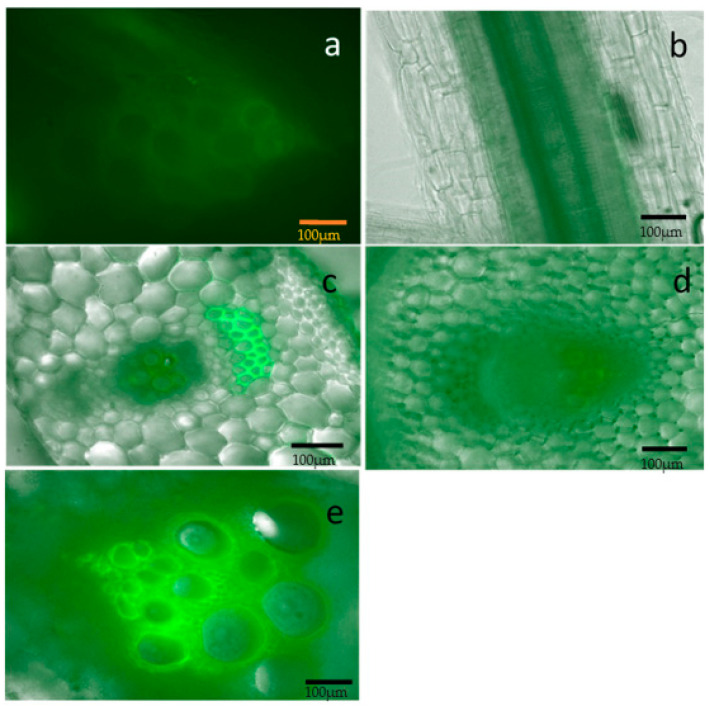
Localization of GFP bacteria in tissues of resistant melon line Ames 13299 at 21 days post inoculation. (**a**): Micrograph of a cross-section of primary root under fluorescence microscope showing GFP-labeled bacteria concentrated in the vascular system. (**b**–**e**): Overlay image of bright field and GFP fluorescence signal of (**b**): secondary root showing the concentration of bacteria in the cortical tissues, (**c**): inoculated leaf petiole, (**d**): petiole topmost leaf at 188 cm above the inoculated leaf and (**e**): stem at 15 cm below inoculated leaf. The presence of bacteria is indicated by the fluorescence. Scale bars = 100 µm.

**Figure 3:**
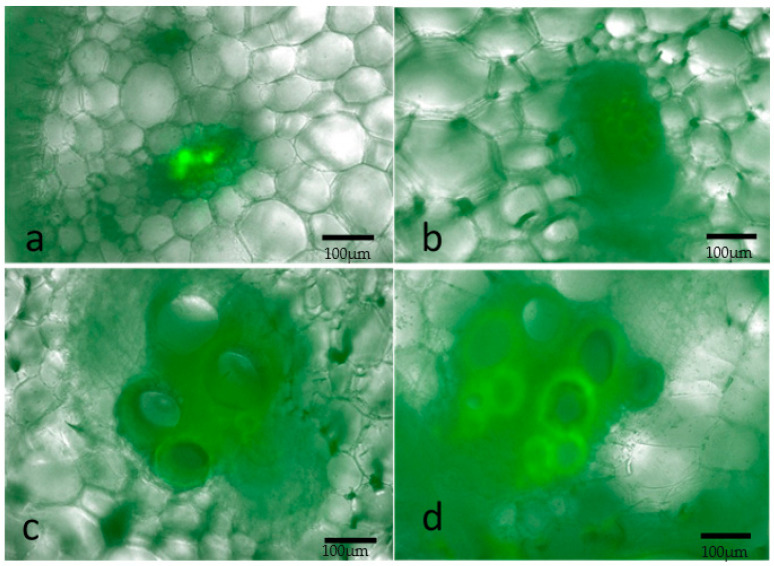
Localization of GFP-labeled bacteria in susceptible line PI 218071 9dpi. Cross-section micrographs of overlay of bright field and GFP fluorescence signal showing GFP-labeled bacteria concentrated in vascular system. (**a**): inoculated leaf petiole, (**b**): topmost leaf petiole at 12 cm above the inoculated leaf, (**c**): stem at 5 cm above the inoculated leaf and (**d**): stem 8 cm below the inoculated leaf. The presence of bacteria is indicated by the fluorescence. Scale bars = 100 µm.

We investigated differences between resistant and susceptible lines in bacterial colonization, movement and location within the plant. The tissues of inoculated plants of the highly resistant melon line Ames 13299, and the highly susceptible line, PI 218071, was examined under a fluorescent microscope. We found that the movement of fluorescently labeled bacterial cells of Hca1-5N was similar in Ames 13299 as compared to PI 218071. Interestingly, we spotted bacteria in the petiole of the topmost leaf, stem and roots in Ames 13299-inoculated plants 21 days post inoculation (dpi) even when the plant was not yet showing any symptoms (Figure 2). Moreover, bacteria were observed throughout the plant in PI 218071, the susceptible line as early as 9 dpi or the day of wilting of inoculated leaf (Figure 3). 

## 3. Discussion

Bacterial wilt causes up to 80% of yield loss in susceptible cultivars of cucurbits and is recognized as an important disease of cucurbits in the United States [7,8]. The management of the disease is mostly based on control of vectors [10], as there is no effective pathogen-based disease management strategy. Once a plant is infected, there is only one outcome, the death of the plant [19,24]. Therefore, sanitation remains the most effective management approach for preventing this disease in the first place. The level of susceptibility of cultivated cucurbits to bacteria wilt differs by cucurbit host species and sometimes cultivar. For instance, watermelon is mostly resistant to bacteria wilt, while cucumber is the most susceptible host followed by melon, squash and pumpkin [9,10].

The United States Department of Agriculture (USDA) has a large germplasm collection of melon accessions from around the globe with wide phenotypic and genotypic diversity, which can be explored efficiently as a source of resistance in breeding programs to enhance various traits including fruit quality and resistance to the myriad diseases afflicting cucurbits [25]. Given the diversity and the size of the collection, we thought that there is a good chance of finding bacterial wilt disease resistance in this collection. Thus, we screened 123 accessions from the USDA collection for resistance against bacteria wilt. We identified four potential melon accessions that can be taken through more vigorous testing including field trials and incorporated into breeding programs. It was not surprising that all the resistance accessions were melon because only five out of the 123 lines screened were cucumber. It is entirely possible that if we had included as many cucumber lines as we did melon, the screen would have yielded some resistant cucumber as well.

There are breeding programs for resistance against various fungal and viral diseases including Fusarium wilt, powdery mildew, cucumber mosaic virus (CMV) and cucurbit yellow stunting disorder virus (CYSDV) in melon (https://cuccap.org/breeding/melon/; accessed on 17 September 2021). Although we do not know of any established breeding programs for bacterial diseases in melon, there are reports of germplasm screening for resistance against bacterial fruit blotch (BFB) disease caused by the seed-transmitted bacterium, *Acidovorax citrulli* [26,27,28]. Recently, Islam et al. [29] have identified an Indel marker associated with BFB resistance in a melon accession. However, we could not find any report of a systematic screening for BW resistance in melons. Although varying levels of resistance have been reported mostly by small scale vendors, no lines of melon have been identified and characterized as resistant to bacteria wilt. Thus, our work represented the first systematic screening of the USDA melon collection against the important bacterial wilt disease. The germplasm accession lines identified in this study will be important in the breeding program for bacterial wilt resistance.

The results from our fluorescent microscopy imaging to determine whether the bacterial colonization was impaired in germplasm accessions with delayed symptom development indicate that the pathogen colonized both host types equally. This is an important observation clearly suggesting that the differences between resistant and susceptible accessions in response to *E. tracheiphila* inoculation may be explained by lack of multiplication of the pathogen in the resistant accessions. However, these results could not rule out the possibility that subtle differences in the colonization rate or bacterial load are responsible for the delayed symptom development in resistant accessions. At the very least, this observation suggests that a lack of colonization may not be responsible for the delayed symptom development in the resistant lines. Our observations are similar to those of Vrisman et al. [30] who observed the presence of bioluminescent *E. tracheiphila* in petioles and main stem of melon following leaf inoculations. They also monitored differences in colonization dynamics of cucurbit host inoculated with bioluminescently labeled *Erwinia tracheiphila*. Our results further confirm the observations of Liu et al. [31] that *E. tracheiphila* also spread downwards to the roots. They also observed that bacterial wilt symptoms are impacted by host age and involve net downward movement of *Erwinia tracheiphila* in muskmelon.

Thus, the microscopic observations in our study indicate that the bacterium was not affected in its ability to colonize both the susceptible and the resistant host. This suggests that the resistant accessions in our experiment do not resist the disease by inhibiting the multiplication or spread of the pathogen. One possible explanation for the lack of symptoms in the resistant host is failure or delay in the pathogen switching from an endophytic to pathogenic state. Indeed, some organisms such as *Xylella fastidiosa*, which is a powerful pathogen in some hosts such as grape, remain harmless endophytes in some other hosts [32,33]. We have initiated a more careful study including inoculum titration to shed more light on any differences between the colonization dynamics of the resistant and susceptible host.

There has been considerable research progress on the genetics of both the pathogen and host species. Draft genome sequences of numerous isolates of the pathogen are now available, and analyses of those genomes have classified the species into three phylogenetic lineages and revealed that there is narrow genetic diversity within the lineages. There is also host specificity within the species with some lineages preferring some host species over others [11]. On the host side, the genome of the double haploid melon line DHL92 is fully sequenced [34] and improvements have been made since then to obtain upgraded versions of melon pseudochromosomes [35,36]. These resources, coupled with the identification of resistance accession in this study, will serve as a foundation for a program to identify the loci associated with the resistance and their eventual incorporation into the cultivated varieties.

## 4. Materials and Methods

### 4.1. Plant Materials and Bacterial Strain

There were 118 melon and five cucumber accessions that were used in the screening tests. Seeds of all the accessions were obtained from USDA germplasm collection. The complete list of the 123 lines and their taxonomic status and geographic origins are provided in Supplemental Table S1 and also available at https://www.ars-grin.gov/ (accessed on 17 September 2021). The *E. tracheiphila* strain used in our screening tests was Hca1-5N [37].

### 4.2. Inoculation, Disease Screening and Statistical Analysis of Data

The screening tests were conducted in two experiments, EXPT1 and EXPT2, in Summer 2019 (May end to July) and Autumn 2019 (September end to November), respectively, in the greenhouse facility of the Tennessee State University. EXPT1 and EXPT2 had 88 and 99 accessions, respectively. Both experiments were carried out in completely randomized design with three replications per accession and one plant per replication. Two seeds of each line were planted in a plastic pot (10.2 cm × 10.2 cm) filled with premium all-purpose potting mix. After germination, the pots were thinned and only one plant was kept per pot. The plants were fertilized once in each experiment by drenching the soil with Sta-Green applied at the recommended rate.

Inoculation was performed when the first true leaf was fully expanded. For inoculum preparation, *E. tracheiphila* strain Hca1-5N was inoculated in nutrient yeast extract (NY) agar plates supplemented with nalidixic acid (25 µg/mL) and incubated at 28 °C for 3 days. The cultures were scraped from agar plates, and a suspension of A_600_ = 0.05 (1 × 10^3^ CFU/mL) was prepared in phosphate buffer. Mechanical inoculation was conducted using a toothbrush-sized stainless steel wire brush following our established protocol [37].

We used three parameters to assess BW resistance of each line including days to wilting of the inoculated leaf (DWIL), days to wilting of the whole plant (DWWP) and days to death of the plant (DDP) (Figure 1).

The data were analyzed with one-way ANOVA using SAS 9.4, with Tukey’s HSD as post hoc test.

### 4.3. Fluorescence Microscopy

We tracked pathogen movement dynamics inside resistant and susceptible melon plants using fluorescence microscopy. For inoculation, the Hca1-5N/pCKD300 was used. The plasmid pCKD300 carries the *gfp* gene under the control of lac promoter and was maintained in nalidixic acid (25 µg/mL) and ampicillin (15 µg/mL)-supplemented NY medium. Microscopic examination was conducted on two melon lines: Ames 13299 and PI 218071, which were the most resistant and susceptible accessions, respectively, in our screening tests. In summer 2020, the two accessions were inoculated with Hca1-5N/pCKD300. Microscopic observations were performed 9 days post inoculation (dpi) and 21 dpi for the susceptible and resistant lines, respectively. Cross-sections were made from different organs of the plants including petioles of inoculated and the topmost leaves, stem (below and above the inoculated leaf) and roots were observed under epifluorescence using Keyence microscope model BZ-710 (Keyence, Itasca, IL, USA).

## Figures and Tables

**Table 1:** Disease severity of melon and cucumber lines inoculated with *E. tracheiphila* Hca1-5N in Summer 2019 and Autumn 2019 screening tests.

	Summer 2019 (Experiment 1)			Autumn 2019 (Experiment 2)			
Accessions	^†^ DWILMean ± SD	^†^ DWWP Mean ± SD	^†^ DDP Mean ± SD	^†^ DWIL Mean ± SD	^†^ DWWP Mean ± SD	^†^ DDP Mean ± SD	* Resistance Classification
Ames 13299	12.67 ± 2.31 ^a^	17.67 ± 6.66 ^a^	21 ± 9.64 ^abc^	6.67 ± 1.15 ^cdefghi^	16 ± 3 ^abcd^	19 ± 1.73 ^abcdef^	H
PI 200814	4.0 ± 0 ^b^	8.67 ± 1.15 ^ghijkl^	11.33 ± 2.31 ^ijkl^	10.33 ± 0.58 ^abc^	14.33 ± 2.31 ^abcdefgh^	20.67 ± 3.21 ^abc^	H
PI 230186	4.0 ± 0 ^b^	12 ± 0 ^cde^	18.0 ± 0 ^bcde^	12.67 ± 8.14 ^ab^	16 ± 6.93 ^abcd^	18.33 ± 7.57 ^abcdefg^	H
PI 370441	4.67 ± 1.15 ^b^	9.67 ± 0.58 ^defghijkl^	11 ± 10.54 ^jkl^	13.33 ± 14.47 ^a^	18 ± 10.44 ^ab^	19.33 ± 9.24 ^abcde^	H
PI 206043 **	4 ± 0 ^b^	12 ± 0 ^cde^	14.67 ± 0.58 ^efghij^	8 ± 0 ^cdefghi^	11.33 ± 1.53 ^efghijklmno^	17.33 ± 2.52 ^abcdefghij^	L
Ames 2830	4.0 ± 0 ^b^	7.33 ± 0.58 ^lk^	10.0 ± 0 ^l^	7.33 ± 1.15 ^cdefghi^	14.67 ± 1.53 ^abcdefg^	15 ± 2 ^defghijklmnop^	L
PI 197891	6.0 ± 2.31 ^b^	10.33 ± 0.58 ^defghij^	14.33 ± 0.58 ^efghijk^	9.67 ± 4.62 ^abcd^	14 ± 3.46 ^bcdefghi^	18 ± 3.46 ^abcdefgh^	L
PI 199097	5.33 ± 2.31 ^b^	8.67 ± 1.15 ^ghijkl^	12.67 ± 2.31 ^ghijkl^	8.33 ± 1.15 ^cdefgh^	13 ± 2 ^cdefghijkl^	16.67 ± 1.53 ^abcdefghijkl^	L
PI 200816	4.0 ± 0 ^b^	9.33 ± 1.15 ^efghijkl^	12.0 ± 2 ^hijkl^	8 ± 1 ^cdefghi^	13 ± 0 ^cdefghijkl^	18 ± 1.73 ^abcdefgh^	L
PI 207660	6.0 ± 2.31 ^b^	9.33 ± 1.15 ^efghijkl^	12.67 ± 2.31 ^ghijkl^	8.33 ± 2.31 ^cdefgh^	13.67 ± 1.15 ^cdefghij^	19 ± 1.73 ^abcdef^	L
PI 211936	4.0 ± 0 ^b^	8.0 ± 0 ^ijkl^	10.0 ± 0 ^l^	6 ± 1.73 ^defghi^	12.33 ± 0.58 ^cdefghijklm^	16.33 ± 0.58 ^abcdefghijkl^	L
PI 251778	4.0 ± 0 ^b^	8.67 ± 1.15 ^ghijkl^	10.67 ± 1.15 ^kl^	7.33 ± 0.58 ^cdefghi^	11.67 ± 3.51 ^efghijklmn^	17 ± 2.65 ^abcdefghijk^	L
PI 255948	4.0 ± 0 ^b^	10.0 ± 0 ^defghijk^	21 ± 0 ^abc^	7 ± 0 ^cdefghi^	8 ± 0 ^nop^	14.33 ± 1.15 ^efghijklmnop^	L
PI 261644	5.33 ± 2.31 ^b^	14 ± 1.73 ^bc^	23 ± 0 ^a^	4.33 ± 0.58 ^i^	5.33 ± 0.58 ^p^	8 ± 0 ^s^	L
PI 277281	4.0 ± 0 ^b^	8.67 ± 1.15 ^ghijkl^	10.67 ± 1.15 ^kl^	6 ± 0 ^defghi^	13.33 ± 0.58 ^cdefghijk^	18 ± 3.46 ^abcdefgh^	L
PI 344068	4.0 ± 0 ^b^	10 ± 0 ^defghijk^	14.67 ± 2.52 ^efghij^	6.67 ± 1.15 ^cdefghi^	12.33 ± 4.04 ^cdefghijklm^	17 ± 5.20 ^abcdefghijk^	L
PI 378558	4.0 ± 0 ^b^	10.0 ± 0 ^defghijk^	21.67 ± 1.15 ^ab^	6 ± 2 ^defghi^	11.67 ± 2.31 ^edfghijklmn^	13.33 ± 2.08 ^ghijklmnopqr^	L
PI 401603	4.0 ± 0 ^b^	10.67 ± 1.15 ^defghi^	19.67 ± 1.15 ^abcd^	6 ± 0 ^defghi^	9.67 ± 1.15 ^jklmno^	11.67 ± 1.15 ^lmnopqrs^	L
Ames 512543	4.0 ± 0 ^b^	7.67 ± 0.58 ^lkj^	11.33 ± 2.31 ^ijkl^	5 ± 1.73 ^ghi^	11 ± 2 ^fghijklmno^	19.67 ± 4.93 ^abcd^	L
PI 500365 **	5 ± 1.73 ^b^	16 ± 3.46 ^ab^	19.67 ± 2.31 ^abcd^	7.33 ± 1.15 ^cdefghi^	14 ± 3 ^bcdefghi^	19 ± 3.61 ^abcdef^	M
PI 344436	6.0 ± 2.31 ^b^	9.67 ± 0.58 ^defghijkl^	12.67 ± 1.15 ^ghijkl^	6 ± 0 ^defghi^	18.33 ± 1.53 ^a^	20.67 ± 1.15 ^abc^	M
PI 378060	4.0 ± 0 ^b^	8.0 ± 0 ^ijkl^	10.0 ± 0 ^l^	8.33 ± 4.04 ^cdefgh^	16 ± 12.12 ^abcd^	17.33 ± 10.97 ^abcdefghij^	M
PI 618838	5.33 ± 2.31 ^b^	7.67 ± 0.58 ^lkj^	10.0 ± 0 ^l^	6 ± 0 ^defghi^	16.33 ± 1.15 ^abc^	21.33 ± 3.79 ^a^	M
Ames 13247 **	4 ± 0 ^b^	10.0 ± 2 ^defghijk^	12.33 ± 2.52 ^ghijkl^	7.33 ± 1.15 ^cdefghi^	10.67 ± 0.58 ^ghijklmno^	13.67 ± 1.15 ^ghijklmnopq^	S
Ames 13251	4.0 ± 0 ^b^	10.0 ± 0 ^defghijk^	12 ± 0 ^hijkl^	8 ± 0 ^cdefghi^	10.67 ± 0.58 ^ghijklmno^	16 ± 3.61 ^bcdefghijklm^	S
Ames 13264	4.0 ± 0 ^b^	10.0 ± 0 ^defghijk^	12.0 ± 0 ^hijkl^	6 ± 2 ^defghi^	7.33 ± 9.06 ^op^	11 ± 3 ^mnopqrs^	S
Ames 13268	4.0 ± 0 ^b^	10.67 ± 1.15 ^defghi^	12.67 ± 1.15 ^ghijkl^	6.33 ± 2.89 ^defghi^	9.33 ± 2.89 ^klmnop^	12 ± 3.46 ^klmnopqrs^	S
Ames 13270	4.0 ± 0 ^b^	10.67 ± 1.15 ^defghi^	12.67± 1.15 ^ghijkl^	6.67 ± 2.31 ^cdefghi^	10.67 ± 0.58 ^ghijklmno^	15 ± 4.58 ^defghijklmnop^	S
Ames 13285	6.0 ± 2.31 ^b^	8.67 ± 1.15 ^ghijkl^	11.33 ± 2.31 ^ijkl^	5 ± 3.61 ^ghi^	9.67 ± 5.13 ^jklmno^	14.67 ± 7.64 ^defghijklmnop^	S
Ames 13305	4.0 ± 0 ^b^	10.0 ± 3.46 ^defghijk^	12.67 ± 4.62 ^ghijkl^	5.33 ± 1.15 ^fghi^	12 ± 2.65 ^defghijklmn^	15 ± 1.73 ^defghijklmnop^	S
Ames 13332	6.0 ± 2.31 ^b^	12 ± 2 ^cde^	17.33 ± 4.93 ^cdef^	8 ± 0 ^cdefghi^	12.33 ± 2.08 ^cdefghijklm^	14.67 ± 4.04 ^defghijklmnop^	S
Ames 19036	4.0 ± 0 ^b^	9.67 ± 2.08 ^defghijkl^	14.33 ± 3.46 ^efghijk^	6.67 ± 1.15 ^cdefghi^	13.33 ± 0.58 ^cdefghijk^	15.67 ± 1.15 ^cdefghijklmn^	S
Ames 20203	4.0 ± 0 ^b^	9.33 ± 1.15 ^efghijkl^	11.33 ± 1.15 ^ijkl^	6 ± 0 ^defghi^	9.67 ± 1.15 ^jklmno^	10.67 ± 2.08 ^nopqrs^	S
Ames 20219	4.0 ± 0 ^b^	7.0 ± 0 ^l^	10.0 ± 0 ^l^	6.67 ± 1.15 ^cdefghi^	9 ± 0 ^lmnop^	13 ± 0 ^hijklmnopqrs^	S
NSL 5648	4.0 ± 0 ^b^	8.0 ± 0 ^ijkl^	10.0 ± 0 ^l^	8 ± 0 ^cdefghi^	9.33 ± 0.58 ^klmnop^	10.33 ± 0.58 ^opqrs^	S
NSL 8521	4.0 ± 0 ^b^	8.0 ± 0 ^ijkl^	10.0 ± 0 ^l^	5.33 ± 1.15 ^fghi^	9 ± 0 ^lmnop^	9 ± 0 ^qrs^	S
PI 193495	5.0 ± 0.73 ^b^	8.0 ± 0 ^ijkl^	10.0 ± 0 ^l^	6 ± 0 ^defghi^	9 ± 0 ^lmnop^	9 ± 0 ^qrs^	S
PI 197077	5.33 ± 2.31 ^b^	8.67 ± 1.15 ^ghijkl^	10.67 ± 1.15 ^kl^	5.33 ± 3.06 ^fghi^	10.33 ± 1.15 ^hijklmno^	13.67 ± 3.51 ^ghijklmnopq^	S
PI 204691	4.0 ± 0 ^b^	8.67 ± 0.58 ^ghijkl^	11.33 ± 1.15 ^ijkl^	7.67 ± 1.15 ^cdefghi^	10.67 ± 1.15 ^ghijklmno^	13 ± 0 ^hijklmnopqrs^	S
PI 210768	4.0 ± 0 ^b^	10 ± 0 ^defghijk^	18.0 ± 3 ^bcde^	7 ± 0 ^cdefghi^	12 ± 0 ^defghijklmn^	16 ± 0 ^bcdefghijklm^	S
PI 211923	4.0 ± 0 ^b^	8.33 ± 0.58 ^hijkl^	11.33 ± 1.15 ^ijkl^	9 ± 1.73 ^bcdef^	11.33 ± 1.15 ^edfghijklmno^	15 ± 1.73 ^defghijklmnop^	S
PI 211946	4.0 ± 0 ^b^	10.0 ± 0 ^defghijk^	12.67 ± 1.15 ^ghijkl^	5 ± 1.73 ^ghi^	8.67 ± 0.58 ^mnop^	11.67 ± 2.31 ^lmnopqrs^	S
PI 211957	4.0 ± 0 ^b^	12 ± 3 ^cde^	17.33 ± 4.73 ^cdef^	6 ± 0 ^defghi^	10 ± 0 ^ijklmno^	11 ± 0 ^mnopqrs^	S
PI 213247	4.0 ± 0 ^b^	11.67 ± 2.89 ^cdef^	16 ± 4.58 ^defg^	4.67 ± 1.15 ^hi^	10.33 ± 2.31 ^hijklmno^	11 ± 2.65 ^mnopqrs^	S
PI 218071	4.0 ± 0 ^b^	8.0 ± 0 ^ijkl^	10.0 ± 0 ^l^	5.33 ± 1.15 ^fghi^	9 ± 0 ^lmnop^	9 ± 0 ^qrs^	S
PI 222098	4.0 ± 0 ^b^	8.33 ± 0.58 ^hijkl^	10.67 ± 1.15 ^kl^	6.67 ± 1.15 ^cdefghi^	9.67 ± 1.15 ^jklmno^	13.33 ± 5.86 ^ghijklmnopqr^	S
PI 223770	4.0 ± 0 ^b^	7.67 ± 0.58 ^lkj^	10.0 ± 0 ^l^	6 ± 0 ^defghi^	9.67 ± 0.58 ^jklmno^	15 ± 1.73 ^defghijklmno^	S
PI 224770	4.0 ± 0 ^b^	8.0 ± 0 ^ijkl^	10.0 ± 0 ^l^	5.33 ± 3.79 ^fghi^	9 ± 1.73 ^lmnop^	12 ± 0 ^klmnopqrs^	S
PI 266932	4.0 ± 0 ^b^	12 ± 0 ^cde^	14.0 ± 0 ^fghijk^	7.33 ± 0.58 ^cdefghi^	10.33 ± 0.58 ^hijklmno^	14 ± 1.73 ^fghijklmnopoq^	S
PI 266942	6.67 ± 2.31 ^b^	10.0 ± 2 ^defghijk^	15.33 ± 3.51 ^efgh^	6 ± 3 ^defghi^	7.33 ± 3.51 ^op^	8.33 ± 3.51 ^rs^	S
PI 277280	4.0 ± 0 ^b^	9.33 ± 2.31 ^efghijkl^	14.67 ± 1.15 ^efghij^	9 ± 1 ^bcdef^	10.67 ± 0.58 ^ghijklmno^	15 ± 1.73 ^defghijklmnop^	S
PI 302446	4.0 ± 0 ^b^	8.0 ± 0 ^ijkl^	10.0 ± 0 ^l^	6 ± 0 ^defghi^	12 ± 5.20 ^defghijklmn^	13.33 ± 5.77 ^ghijklmnopqr^	S
PI 344345	4.0 ± 0 ^b^	9.33 ± 1.15 ^efghijkl^	15.33 ± 5.51 ^efgh^	5.33 ± 1.15 ^fghi^	9.33 ± 0.58 ^klmnop^	13.33 ± 0.58 ^ghijklmnopqr^	S
PI 357756	6.33 ± 1.15 ^b^	10.0 ± 0 ^defghijk^	14.0 ± 1.73 ^fghijk^	5 ± 3.61 ^ghi^	9.33 ± 6.35 ^klmnop^	12.33 ± 3.79 ^jklmnopqrs^	S
PI 357783	6.0 ± 0 ^b^	10.0 ± 0 ^defghijk^	12.0 ± 0 ^hijkl^	6 ± 0 ^defghi^	10.33 ± 1.15 ^hijklmno^	14.33 ± 0.58 ^efghijklmnop^	S
PI 401600	4.0 ± 0 ^b^	9.33 ± 1.15 ^efghijkl^	15.33 ± 4.62 ^efgh^	6 ± 0 ^defghi^	11.67 ± 1.15 ^efghijklmn^	14 ± 1 ^fghijklmnop^	S
PI 419220	4.0 ± 0 ^b^	11.32 ± 2.31 ^cdefg^	15 ± 1.73 ^efghi^	6 ± 0 ^defghi^	9 ± 0 ^lmnop^	13.33 ± 0.58 ^ghijklmnopqr^	S
PI 482400	4.0 ± 0 ^b^	7.0 ± 0 ^l^	10.0 ± 0 ^l^	6 ± 0 ^defghi^	10.67 ± 2.89 ^ghijklmno^	15.33 ± 5.13 ^defghijklmno^	S
PI 502329	5.67 ± 2.31 ^b^	12.33 ± 4.93 ^cd^	15 ± 5.20 ^efghi^	6 ± 3.46 ^defghi^	11 ± 0 ^fghijklmno^	14.33 ± 2.31 ^efghijklmnop^	S
PI 505611	6.67 ± 2.31 ^b^	9.33 ± 2.31 ^efghijkl^	12.0 ± 2 ^hijkl^	6 ± 0 ^defghi^	9.67 ± 1.15 ^jklmno^	12 ± 2.65 ^klmnopqrs^	S
PI 601164	4.67 ± 1.15 ^b^	8.67 ± 1.15 ^ghijkl^	11.33 ± 2.31 ^ijkl^	8 ± 0 ^cdefghi^	9 ± 0 ^lmnop^	13 ± 1.73 ^hijklmnopqrs^	S
PI 614161	4.0 ± 0 ^b^	8.0 ± 0 ^ijkl^	10.0 ± 0 ^l^	8 ± 0 ^cdefghi^	8.67 ± 0.58 ^mnop^	10 ± 1 ^pqrs^	S
PI 618819	4.0 ± 0 ^b^	8.67 ± 1.15 ^ghijkl^	10.67 ± 1.15 ^kl^	8.67 ± 1.15 ^cdefg^	11.67 ± 1.15 ^efghijklm^	14.33 ± 2.31 ^efghijklmnop^	S
PI 505598	4.0 ± 0 ^b^	8.0 ± 0 ^ijkl^	10.0 ± 0 ^l^	6.33 ± 2.89 ^defghi^	10.33 ± 2.31 ^hijklmno^	15.67 ± 4.62 ^cdefghijklmn^	S

* S = susceptible (low value for all three parameters); L = low resistance (high in any one parameter from either experiment); M = medium resistance (high in any two parameters from either experiment); H = high resistance (high in all three parameters in either experiment). ** Cucumber accessions. All the rest are melon accessions. **^†^** DWIL = days to wilting of inoculated leaf; DWWP = days to wilting of whole plant; DDP = days to death of the plant. Means followed by the same letters are not significantly different from each other. An accession is considered resistant for a particular parameter if its mean is followed by the letter “a” using Tukey’s HSD test.

**Table 2:** Mean disease scores of melon and cucumber lines inoculated with *E. tracheiphila* Hca1-5N in one season screening test (Summer 2019 or Autumn 2019) *.

Accessions	^†^ DWIL Mean ± SD	^†^ DWWP Mean ± SD	^†^ DDP Mean ± SD	Experiment	* Resistance Classification
PI 229309 **	8 ± 0 ^cdefghi^	12.33 ± 2.08 ^cdefghijklm^	14.33 ± 3.51 ^efghijklmnop^	Autumn 2019	S
PI 207659	4.0 ± 0 ^b^	14 ± 0 ^bc^	21 ± 0 ^abc^	Summer 2019	L
Ames 13248	5.33 ± 4.04 ^fghi^	9 ± 6.24 ^lmnop^	17.33 ± 3.51 ^abcdefghij^	Autumn 2019	L
Ames 13321	8 ± 0 ^cdefghi^	14 ± 5.20 ^bcdefghi^	19 ± 1.73 ^abcdef^	Autumn 2019	L
PI 207661	7.67 ± 0.58 ^cdefghi^	13 ± 0 ^cdefghijkl^	17 ± 0 ^abcdefghijk^	Autumn 2019	L
PI 211948	7 ± 0 ^cdefghi^	12 ± 0 ^defghijklmn^	18 ± 2 ^abcdefgh^	Autumn 2019	L
PI 236355	9.33 ± 2.08^bcde^	14 ± 1.73 ^bcdefghi^	21 ± 4.36 ^ab^	Autumn 2019	L
PI 292312	6 ± 0 ^defghi^	13.33 ± 0.58 ^cdefghijk^	16.67 ± 2.89 ^abcdefghijkl^	Autumn 2019	L
PI 403994	7.33 ± 1.53 ^cdefghi^	11.33 ± 0.58 ^efghijklmno^	19.67 ± 4.51 ^abcd^	Autumn 2019	L
PI 211016	7 ± 0 ^cdefghi^	15.33 ± 2.52 ^abcde^	21 ± 3.46 ^ab^	Autumn 2019	M
PI 502328	6 ± 0 ^defghi^	15 ± 2 ^abcdef^	17.67 ± 3.51 ^abcdefghi^	Autumn 2019	M
Ames 2822	4.0 ± 0 ^b^	8.0 ± 0 ^ijkl^	10.0 ± 0 ^l^	Summer 2019	S
Ames 2824	4.0 ±0 ^b^	8.0 ± 0 ^ijkl^	10.0 ± 0 ^l^	Summer 2019	S
Ames 2826	4.0 ± 0 ^b^	10.0 ± 0 ^defghijk^	12.0 ± 0 ^hijkl^	Summer 2019	S
Ames 13261	4.0 ± 0 ^b^	9.0 ± 1.73 ^fghijkl^	12.67 ± 1.15 ^ghijkl^	Summer 2019	S
Ames 18738	4.0 ± 0 ^b^	10.0 ± 0 ^defghijk^	15 ± 0 ^efghi^	Summer 2019	S
PI 183676	4.0 ± 0 ^b^	9.67 ± 1.53 ^defghijkl^	16 ± 6.56 ^defg^	Summer 2019	S
PI 211922	4.0 ± 0 ^b^	8.0± ^ijkl^	10.0 ± 0 ^l^	Summer 2019	S
PI 218070	4.0 ± 0 ^b^	11 ± 1 ^defgh^	15 ± 0 ^efghi^	Summer 2019	S
PI 223636	4.0 ± 0 ^b^	7.67 ± 0.58 ^lkj^	10.0 ± 0 ^l^	Summer 2019	S
PI 26443	4.67 ± 1.15 ^b^	9.33 ± 1.15 ^efghijkl^	12.0 ± 2 ^hijkl^	Summer 2019	S
PI 244713	4.0 ± 0 ^b^	10.0 ± 0 ^defghijk^	14.0 ± 0 ^fghijk^	Summer 2019	S
PI 266946	4.0 ± 0 ^b^	10.0 ± 2 ^defghijk^	13.33 ± 1.15 ^ghijkl^	Summer 2019	S
PI 267083	4.0 ± 0 ^b^	11.33 ± 2.31 ^cdefg^	15 ± 5.20 ^efghi^	Summer 2019	S
PI 271329	4.0 ± 0 ^b^	7.0 ± 0 ^l^	10.0 ± 0 ^l^	Summer 2019	S
PI 344318	4.0 ± 0 ^b^	8.33 ± 0.58 ^hijkl^	10.67 ± 1.15 ^kl^	Summer 2019	S
PI 370021	4.0 ± 0 ^b^	8.0 ± 0 ^ijkl^	10.0 ± 0 ^l^	Summer 2019	S
PI 401655	4.0 ± 0 ^b^	10.0 ± 0 ^defghijk^	13.67 ± 1.53 ^fghijkl^	Summer 2019	S
PI 504527	4.0 ± 0 ^b^	8.0 ± 0 ^ijkl^	10.0 ± 0 ^l^	Summer 2019	S
PI 614159	4.0 ± 0 ^b^	10.67 ± 2.31 ^defghi^	12.67 ± 2.31 ^ghijkl^	Summer 2019	S
Ames 13302	4.0 ± 0 ^b^	8.67 ± 1.15 ^ghijkl^	11.33 ± 2.31 ^ijkl^	Summer 2019	S
Ames 13304	4.0 ± 0 ^b^	8.0 ± 0 ^ijkl^	10.0 ± 0 ^l^	Summer 2019	S
Ames 13317	4.0 ± 0 ^b^	8.67 ± 1.15 ^ghijkl^	10.67 ± 1.15 ^kl^	Summer 2019	S
Ames 13318	5.33 ± 2.31 ^b^	8.0 ± 0 ^ijkl^	10.0 ± 0 ^l^	Summer 2019	S
Ames 13257 **	6 ± 0 ^defghi^	9.67 ± 1.15 ^jklmno^	14.67 ± 2.08 ^defghijklmnop^	Autumn 2019	S
Ames 13292	6.67 ± 1.15 ^cdefghi^	12 ± 1.73 ^defghijklmn^	13 ± 1.73 ^hijklmnopqrs^	Autumn 2019	S
Ames 13295	8 ± 0 ^cdefghi^	12 ± 1.73 ^defghijklmn^	15.67 ± 4.04 ^cdefghijklmn^	Autumn 2019	S
Ames 13303	6 ± 0 ^defghi^	10.33 ± 2.31 ^hijklmno^	14.33 ± 2.31 ^efghijklmnop^	Autumn 2019	S
Ames 13319	8 ± 0 ^cdefghi^	12 ± 1.73 ^defghijklmn^	16 ± 1.73 ^bcdefghijklm^	Autumn 2019	S
Ames 13325	5.67 ± 2.52 ^efghi^	12.67 ± 2.31 ^cdefghijklm^	15.33 ± 2.52 ^defghijklmno^	Autumn 2019	S
Ames 13337	6 ± 0 ^defghi^	9.67 ± 1.15 ^jklmno^	14 ± 0 ^fghijklmnopq^	Autumn 2019	S
PI 210541	8.33 ± 0.58 ^cdefgh^	12 ± 0 ^defghijklmn^	16 ± 0 ^bcdefghijklm^	Autumn 2019	S
PI 212639	7.33 ± 1.15 ^cdefghi^	10.33 ± 0.58 ^hijklmno^	13 ± 0 ^hijklmnopqrs^	Autumn 2019	S
PI 229750	7.67 ± 0.58 ^cdefghi^	9 ± 1 ^lmnop^	13.33 ± 2.31 ^ghijklmnopqr^	Autumn 2019	S
PI 266943	7.33 ± 0.58 ^cdefghi^	12 ± 0 ^defghijklmn^	15 ± 0 ^defghijklmnop^	Autumn 2019	S
PI 319217	6 ± 0 ^defghi^	10.33 ± 0.58 ^hijklmno^	13.33 ± 0.58 ^ghijklmnopqr^	Autumn 2019	S
PI 319218	6 ± 0 ^defghi^	10 ± 1.73 ^ijklmno^	12.33 ± 2.89 ^jklmnopqrs^	Autumn 2019	S
PI 344346	5.33 ± 2.31 ^fghi^	10.33 ± 1.15 ^hijklmno^	13.33 ± 0.58 ^ghijklmnopqr^	Autumn 2019	S
PI 355715	6 ± 0 ^defghi^	10 ± 1 ^ijklmno^	15.67 ± 1.15 ^cdefghijklmn^	Autumn 2019	S
PI 357758	6 ± 0 ^defghi^	12.33 ± 1.15 ^cdefghijklm^	15.67 ± 1.15 ^cdefghijklmn^	Autumn 2019	S
PI 378059	6 ± 2 ^defghi^	9.33 ± 6.35 ^klmnop^	10.33 ± 2.31 ^opqrs^	Autumn 2019	S
PI 391574	6.67 ± 2.31 ^cdefghi^	12 ± 1.73 ^defghijklmn^	13 ± 1.73 ^hijklmnopqrs^	Autumn 2019	S
PI 406737	7.67 ± 1.15 ^cdefghi^	10.33 ± 0.58 ^hijklmno^	12 ± 0 ^klmnopqrs^	Autumn 2019	S
PI 420146	6 ± 0 ^defghi^	9 ± 0 ^lmnop^	12.33 ± 2.89 ^jklmnopqrs^	Autumn 2019	S
PI 482396	6 ± 0 ^defghi^	12.67± 4.73 ^cdefghijklm^	13.33 ± 0.58 ^ghijklmnopqr^	Autumn 2019	S
PI 482397	6.67 ± 1.15 ^cdefghi^	9.33 ± 0.58 ^klmnop^	13.33 ± 0.58 ^ghijklmnopqr^	Autumn 2019	S
PI 482398	7.33 ± 4.04 ^cdefghi^	9.67 ± 2.89 ^jklmno^	14.33 ± 2.31 ^efghijlkmnop^	Autumn 2019	S
PI 505612	8 ± 0 ^cdefghi^	9 ± 0 ^lmnop^	12.67 ± 2.31 ^ijklmnopqrs^	Autumn 2019	S
PI 512442	6 ± 0 ^defghi^	9 ± 0 ^lmnop^	13.67 ± 3.06 ^ghijklmnopq^	Autumn 2019	S

* S = susceptible (low value for all parameters); L = low resistance (high in any one parameter from either experiments); M = medium resistance (high in any two parameters from either experiments). ** Cucumber accession. All the rest are melon lines. **^†^** DWIL = days to wilting of inoculated leaf; DWWP = days to wilting of whole plant; DDP = days to death of the plant. Means followed by the same letters are not significantly different from each other. An accession is considered resistant for a particular parameter if its mean is followed by the letter “a” using Tukey’s HSD test.

**Table 3:** Summary of disease ratings and resistance classifications of *Cucumis* accessions.

Resistance Level	Number of Accessions Screened in Both Experiments	Number of Accessions Screened in One Experiment	Total
Susceptible (S)	41	49	90
Low (L)	15	8	23
Medium (M)	4	2	6
High (H)	4	0	4
Total	64	59	123

## Data Availability

Please contact the corresponding author.

## Supplementary Materials

The following are available online at https://www.mdpi.com/article/10.3390/plants10091972/s1. Table S1: List of melon accessions from USDA germplasm collection used in the study.
