# Supplementary material for: Identification of Bacterial Wilt (Erwinia tracheiphila) Resistances in USDA Melon Collection
Source: Plants (Basel). 2021 Sep 21;10(9):1972. doi: 10.3390/plants10091972 (PMC8473077; doi:10.3390/plants10091972)
Supplement: Supplementary file 1 [file plants-10-01972-s001.zip › plants-1380419-supplementary.pdf]

Supplementary Material

Table S1. List of melon accessions from USDA germplasm collection used in this study *.

#	Accessions	Taxonomic status	Geographic origin
1	Ames 13247	*Cucumis sativus*	Spain, Jaen
2	Ames 13257	*Cucumis sativus*	Spain, Jaen
3	PI 206043	*Cucumis sativus* var. *sativus*	Puerto Rico
4	PI 229309	*Cucumis sativus* var. *sativus*	Iran, Kerman
5	PI 500365	*Cucumis sativus* var. *sativus*	Zambia, Copperbelt
6	Ames 2822	*Cucumis melo* subsp. *melo*	United States, California
7	Ames 2824	*Cucumis melo* subsp. *melo*	United States, California
8	Ames 2826	*Cucumis melo* subsp. *melo*	United States, California
9	Ames 2830	*Cucumis melo* subsp. *melo*	United States, California
10	Ames 13248	*Cucumis melo* subsp. *melo*	Spain, Jaen
11	Ames 13251	*Cucumis melo* subsp. *melo*	Spain, Jaen
12	Ames 13261	*Cucumis melo* subsp. *melo*	Spain, Jaen
13	Ames 13264	*Cucumis melo* subsp. *melo*	Spain, Cadiz
14	Ames 13268	*Cucumis melo* subsp. *melo*	Spain, Balearic Islands
15	Ames 13270	*Cucumis melo* subsp. *melo*	Spain, Canary Islands
16	Ames 13285	*Cucumis melo* subsp. *melo*	Spain, Gerona
17	Ames 13292	*Cucumis melo* subsp. *melo*	Spain, Albacete
18	Ames 13295	*Cucumis melo* subsp. *melo*	Spain, Cuenca
19	Ames 13299	*Cucumis melo* subsp. *melo*	Spain, Badajoz
20	Ames 13303	*Cucumis melo* subsp. *melo*	Spain, Murcia
21	Ames 13305	*Cucumis melo* subsp. *melo*	Spain, Murcia
22	Ames 13319	*Cucumis melo* subsp. *melo*	Spain, Murcia
23	Ames 13321	*Cucumis melo* subsp. *melo*	Spain, Alicante
24	Ames 13325	*Cucumis melo* subsp. *melo*	Spain, Alicante
25	Ames 13332	*Cucumis melo* subsp. *melo*	Spain, Alicante
26	Ames 13337	*Cucumis melo*	Spain, Jaen
27	Ames 18738	*Cucumis melo* subsp. *melo*	United States, Colorado
28	Ames 19036	*Cucumis melo* subsp. *melo*	Kazakhstan, Alma-Ata
29	Ames 20203	*Cucumis melo* subsp. *melo*	India
30	Ames 20219	*Cucumis melo* subsp. *melo*	United States, New York
31	NSL 5648	*Cucumis melo* subsp. *melo*	United States, Colorado
32	NSL 8521	*Cucumis melo* subsp. *melo*	United States, Michigan
33	PI 183676	*Cucumis melo* subsp. *melo*	Turkey, Trabzon
34	PI 193495	*Cucumis melo* subsp. *melo*	Ethiopia, Shewa
35	PI 197077	*Cucumis melo* subsp. *melo*	Spain
36	PI 197891	*Cucumis melo* subsp. *melo*	United States, Virginia
37	PI 199097	*Cucumis melo* subsp. *melo*	Iran, Tehran
38	PI 200814	*Cucumis melo* subsp. *melo*	Myanmar, Mandalay
39	PI 200816	*Cucumis melo* subsp. *melo*	Myanmar, Mandalay
40	PI 204691	*Cucumis melo* subsp. *melo*	Turkey
41	PI 207659	*Cucumis melo* subsp. *melo*	Morocco
42	PI 207660	*Cucumis melo* subsp. *melo*	Morocco
43	PI 207661	*Cucumis melo* subsp. *melo*	Morocco
44	PI 210541	*Cucumis melo* subsp. *melo*	India, Meghalaya
45	PI 210768	*Cucumis melo* subsp. *melo*	Turkey, Ankara
46	PI 211016	*Cucumis melo* subsp. *melo*	Afghanistan, Baghlan
47	PI 211922	*Cucumis melo* subsp. *melo*	Iran, Tehran
48	PI 211923	*Cucumis melo* subsp. *melo*	Iran, East Azerbaijan

49	PI 211936	*Cucumis melo* subsp. *melo*	Iran, Mazandaran
50	PI 211946	*Cucumis melo* subsp. *melo*	Iran, Tehran
51	PI 211948	*Cucumis melo* subsp. *melo*	Iran, Tehran
52	PI 211957	*Cucumis melo* subsp. *melo*	Iran, Kerman
53	PI 212639	*Cucumis melo* subsp. *melo*	Iran
54	PI 213247	*Cucumis melo* subsp. *melo*	India, Gujarat
55	PI 218070	*Cucumis melo* subsp. *melo*	Pakistan, Punjab
56	PI 218071	*Cucumis melo* subsp. *melo*	Pakistan, North-West Frontier
57	PI 222098	*Cucumis melo* subsp. *melo*	Afghanistan, Kabul
58	PI 223636	*Cucumis melo* subsp. *melo*	Iran, Gilan
59	PI 223770	*Cucumis melo* subsp. *melo*	Afghanistan, Badakhshan
60	PI 224770	*Cucumis melo* subsp. *melo*	Africa
61	PI 26443	*Cucumis melo* subsp. *melo*	Israel
62	PI 229750	*Cucumis melo* subsp. *melo*	Iran, Mazandaran
63	PI 230186	*Cucumis melo* subsp. *melo*	Iran
64	PI 236355	*Cucumis melo* subsp. *melo*	United Kingdom, England
65	PI 244713	*Cucumis melo* subsp. *melo*	United States, Connecticut
66	PI 251778	*Cucumis melo* subsp. *melo*	Soviet Union, Former
67	PI 255948	*Cucumis melo* var. *cantalupo*	Germany
68	PI 261644	*Cucumis melo* subsp. *melo*	Netherlands, Gelderland
69	PI 266932	*Cucumis melo* subsp. *melo*	Japan
70	PI 266942	*Cucumis melo* var. *cantalupo*	United Kingdom, England
71	PI 266943	*Cucumis melo* var. *cantalupo*	France
72	PI 266946	*Cucumis melo* var. *cantalupo*	France
73	PI 267083	*Cucumis melo* subsp. *melo*	Turkmenistan
74	PI 271329	*Cucumis melo* subsp. *melo*	India, Maharashtra
75	PI 277280	*Cucumis melo* subsp. *melo*	India
76	PI 277281	*Cucumis melo* subsp. *melo*	India
77	PI 292312	*Cucumis melo* subsp. *melo*	Uzbekistan
78	PI 302446	*Cucumis melo* subsp. *melo*	India, Delhi
79	PI 319217	*Cucumis melo* subsp. *melo*	Egypt
80	PI 319218	*Cucumis melo* subsp. *melo*	Egypt
81	PI 344068	*Cucumis melo* subsp. *melo*	Turkey, Gaziantep
82	PI 344318	*Cucumis melo* subsp. *melo*	Turkey
83	PI 344345	*Cucumis melo* subsp. *melo*	Turkey, Tunceli
84	PI 344346	*Cucumis melo* subsp. *melo*	Turkey, Antalya
85	PI 344436	*Cucumis melo* subsp. *melo*	Iran, Fars
86	PI 355715	*Cucumis melo* subsp. *melo*	Israel
87	PI 357756	*Cucumis melo* subsp. *melo*	Macedonia
88	PI 357758	*Cucumis melo* subsp. *melo*	Macedonia
89	PI 357783	*Cucumis melo* subsp. *melo*	Macedonia
90	PI 370021	*Cucumis melo* subsp. *melo*	India
91	PI 370441	*Cucumis melo* subsp. *melo*	Macedonia
92	PI 378059	*Cucumis melo* subsp. *melo*	Japan
93	PI 378060	*Cucumis melo* subsp. *melo*	Japan
94	PI 378558	*Cucumis melo* subsp. *melo*	Afghanistan, Balkh
95	PI 391574	*Cucumis melo* subsp. *melo*	China, Shaanxi
96	PI 401600	*Cucumis melo* subsp. *melo*	Spain
97	PI 401603	*Cucumis melo* subsp. *melo*	Spain
98	PI 401655	*Cucumis melo* subsp. *melo*	Spain
99	PI 403994	*Cucumis melo* subsp. *melo*	Colombia, Tolima
100	PI 406737	*Cucumis melo* subsp. *agrestis*	Costa Rica, Puntarenas
101	PI 419220	*Cucumis melo* subsp. *melo*	Poland

102	PI 420146	*Cucumis melo* subsp. *melo*	United States, Arizona
103	PI 482396	*Cucumis melo* subsp. *melo*	Zimbabwe
104	PI 482397	*Cucumis melo* subsp. *melo*	Zimbabwe
105	PI 482398	*Cucumis melo* subsp. *melo*	Zimbabwe
106	PI 482400	*Cucumis melo* subsp. *melo*	Zimbabwe
107	PI 502328	*Cucumis melo* subsp. *melo*	Russian Federation, Kalmykia
108	PI 502329	*Cucumis melo* subsp. *melo*	Ukraine
109	PI 504527	*Cucumis melo* subsp. *melo*	India, Karnataka
110	PI 505611	*Cucumis melo*	Zambia, Southern
111	PI 505612	*Cucumis melo*	Zambia, Southern
112	PI 601164	*Cucumis melo* subsp. *melo*	United States, Alabama
113	PI 614159	*Cucumis melo* subsp. *melo*	India, Rajasthan
114	PI 614161	*Cucumis melo* subsp. *melo*	India, Rajasthan
115	PI 618819	*Cucumis melo* subsp. *melo*	China
116	PI 505598	*Cucumis metuliferus*	Zambia, North-Western
117	Ames 13302	*Cucumis melo* subsp. *melo*	Spain, Murcia
118	Ames 13304	*Cucumis melo* subsp. *melo*	Spain, Murcia
119	Ames 13317	*Cucumis melo* subsp. *melo*	Spain, Murcia
120	Ames 13318	*Cucumis melo* subsp. *melo*	Spain, Murcia
121	PI 512442	*Cucumis melo* subsp. *melo*	Spain, Malaga
122	Ames 512543	*Cucumis melo* subsp. *melo*	Spain, Alicante
123	PI 618838	*Cucumis melo* subsp. *melo*	China, Xinjiang

* Additional information of the accessions is available at https://www.ars-grin.gov/. accessed 17 Sept, 2021
